# Feasibility study of home-based rehabilitation training for language disorders in infants aged 0–1 year

**DOI:** 10.3389/fped.2026.1760426

**Published:** 2026-03-27

**Authors:** Ruihua Kang, Yanjun Wang, Jingli Nie, Jingjiao Wang, Jing Wu

**Affiliations:** 1 Department of Outpatient, Jiangxi Provincial Children’s Hospital-The Affiliated Children’s Hospital of Nanchang Medical College, Nanchang, Jiangxi, China; 2 Department of Traditional Chinese Medicine, Jiangxi Provincial Children’s Hospital-The Affiliated Children’s Hospital of Nanchang Medical College, Nanchang, Jiangxi, China; 3 Department of Rehabilitation Medicine, Jiangxi Provincial Children’s Hospital-The Affiliated Children’s Hospital of Nanchang Medical College, Nanchang, Jiangxi, China

**Keywords:** caregiver training, early intervention, home-based rehabilitation training, infants, language development delay

## Abstract

**Objective:**

Delayed language development in infants under one year of age can lead to persistent developmental consequences. The language of infants under one year primarily develops through face-to-face communication with their parents, such as spontaneous smiling and eye contact with their mother, which fosters language communication and promotes development. This motivates us to explore home-based alternatives. This study aims to assess the feasibility and preliminary effects of a home-based language rehabilitation program for infants aged 0–1, implemented by caregivers.

**Methods:**

A retrospective study was conducted from May 2021 to March 2023 at Jiangxi Children's Hospital to evaluate the effectiveness of speech therapy interventions. A total of 195 cases were included, of which 83 cases were assigned to the experimental group. The experimental group received regular training based on standardized speech rehabilitation training therapy protocols, specifically utilizing a home-based model in which professional speech rehabilitation training instructors provided guidance. This approach focused on enhancing the use of sign language and its integration into the home environment. The control group consisted of 112 cases, who received conventional speech therapy services provided by specialized speech therapists. The primary outcomes assessed included the improvement in speech and language skills, as well as the progression of language acquisition in both groups. Statistical analyses were performed to compare the effectiveness of the two approaches.

**Results:**

At the 6-month follow-up, the proportion of infants in the home training group who reached age-appropriate language milestones was significantly higher (72.3% vs. 48.2%, *p* < 0.01). The caregiver adherence rate was over 85%, and 91.3% of participants expressed high satisfaction with the intervention. No complications related to the intervention were reported.

**Conclusion:**

During periods of disruption in the healthcare system, home-based language rehabilitation is a feasible and effective method for managing early language delays in infants. Further prospective studies are needed to confirm its long-term benefits.

## Introduction

The first year of life is a foundational period for language development, during which infants acquire basic auditory processing, vocalization, and early communicative behaviors. Any disruption in this sensitive phase—whether due to biological, environmental, or socio-cultural factors—can result in persistent language delays, which are closely linked to subsequent deficits in cognitive ability, emotional regulation, academic performance, and social integration (1–3). Therefore, early detection and timely rehabilitation for infants with signs of delayed language development are essential to prevent long-term neurodevelopmental consequences.

Traditionally, language rehabilitation for infants has relied on in-hospital services led by professional speech-language therapists. However, this treatment is a long-term process, and parents' deep concerns about the risk of hospital-acquired infections have significantly hindered the accessibility of such services (4–6). As a result, an urgent need emerged for alternative intervention models that could ensure continuity of early rehabilitation while minimizing hospital visits and maintaining infection control. Home-based rehabilitation models—especially those involving parent-mediated training under professional supervision—have gained increasing attention as a practical alternative. These approaches are designed to empower caregivers to deliver structured, evidence-based language stimulation activities within the familiar and low-stress environment of the home. Studies in older children have demonstrated the effectiveness of caregiver-led interventions in improving speech and language outcomes, enhancing parent–child interaction quality, and increasing therapy adherence (7–11). However, for infants aged 0–12 months, there remains limited research on the implementation feasibility, caregiver compliance, and developmental efficacy of home-based language intervention programs.

In the present study, the term “language disorder” is used descriptively to refer to early language developmental delay and increased risk for later developmental language disorder (DLD), rather than a formal clinical diagnosis as defined by DSM-5. Given the young age of the study population (0–12 months), diagnostic categorization is neither appropriate nor intended. Early social communication behaviors, such as eye contact, social smiling, and caregiver–infant interaction, are foundational for language learning during infancy. However, reduced social communication at this age represents a non-specific developmental risk marker and does not necessarily predict later language disorder. Such early difficulties may be associated with a range of neurodevelopmental trajectories, including but not limited to DLD or autism spectrum disorder (ASD). Accordingly, the present study focuses on early functional risk and intervention responsiveness rather than diagnostic outcomes.

To address this gap, we conducted a retrospective cohort study to examine the feasibility and preliminary outcomes of a structured home-based rehabilitation model for infants with language delay. Between May 2021 and March 2023, 195 infants aged under one year were enrolled and categorized into an intervention group (*n* = 83) that received structured home-based language training and a control group (*n* = 112) that received routine follow-up and general developmental advice. The primary aim was to assess changes in language developmental scores over time, while secondary outcomes included caregiver adherence, satisfaction, and frequency of medical reconsultation. Through this investigation, we aim to provide evidence to support scalable, family-centered rehabilitation pathways—especially relevant in pandemic conditions and for families in resource-limited or rural settings.

## Materials and methods

### Study design and participants

**Figure 1:**
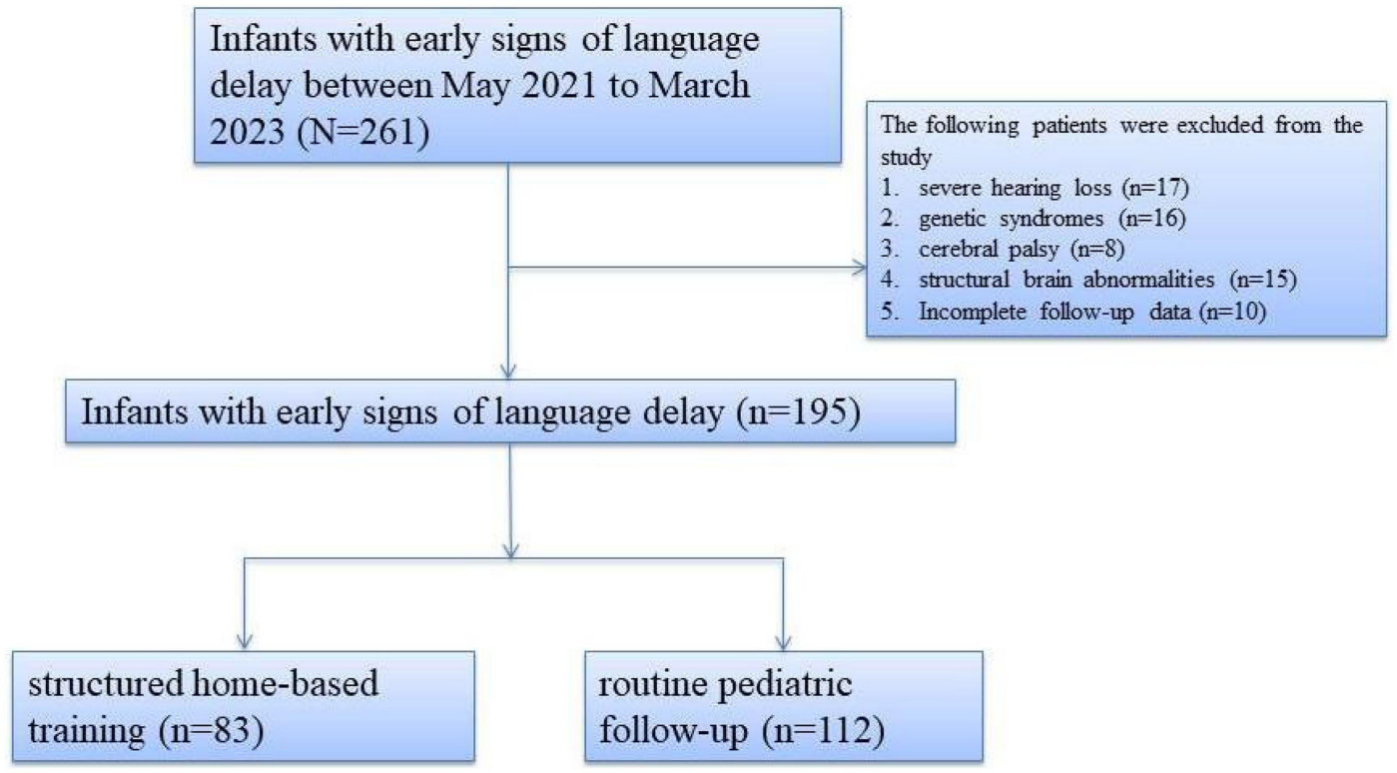
Flowchart of patient selection and group allocation.

This study represents a retrospective analysis of a prospectively followed clinical cohort conducted at the Department of Developmental Rehabilitation, Jiangxi Children's Hospital between May 2021 and March 2023. Infants were enrolled and followed longitudinally during routine clinical care, and all analyses were performed after completion of follow-up (Figure 1). The study aimed to evaluate the feasibility and effectiveness of a home-based language rehabilitation program among infants aged 0–12 months diagnosed with early language delay. A total of 195 infants were included, of whom 83 received structured home-based intervention (intervention group) and 112 infants who received only conventional training, (control group). Eligible participants met the following inclusion criteria: age under 12 months at enrollment, diagnosis of language developmental delay confirmed using standardized tools such as the Gesell Developmental Schedules or CNBS-R2016, and the availability of a primary caregiver willing and able to participate in training and follow-up. Exclusion criteria included severe hearing loss, genetic syndromes, cerebral palsy, structural brain abnormalities, or incomplete follow-up data. Accordingly, the included infants largely represent a population with early language delay and an increased risk for developmental language disorder (DLD), rather than language delay attributable to identifiable medical conditions. Ethical approval was obtained from the hospital's ethics committee (JXSETYY-YXKY-20250143).

### Intervention delivery and format

Baseline and follow-up developmental assessments were conducted in person at the outpatient clinic by trained clinicians using standardized assessment tools. The initial caregiver training session (approximately 90 min) was also delivered face-to-face. During the home-based intervention period, caregivers received ongoing supervision, guidance, and feedback remotely via a secure WeChat-based platform, while daily intervention activities were implemented by caregivers in the home environment.

### Intervention: home-based language rehabilitation protocol

Infants in the intervention group received a structured, caregiver-mediated early language stimulation program designed for infants aged 0–12 months. This program was not a formal sign language therapy, but aimed to enhance early communicative behaviors through daily parent–infant interaction within naturalistic home settings.

The intervention protocol was developed by a multidisciplinary team comprising speech therapists, pediatricians, and rehabilitation nurses. The intervention was delivered using a hybrid model, combining an initial in-person caregiver training session with ongoing remote supervision; no home visits were conducted. Upon enrollment, caregivers participated in a face-to-face training session lasting approximately 90 min, during which intervention principles, procedures, and expected caregiver roles were introduced.

During the training session, caregivers were instructed in age-appropriate language stimulation strategies tailored to the infant's developmental level. Core components of the intervention included responsive vocal interaction, turn-taking, naming routines, object labeling during daily activities, and the use of developmentally appropriate gestures (e.g., pointing and symbolic gestures) to support communicative intent prior to the emergence of spoken language.

Caregivers were then assigned structured home practice tasks and instructed to implement the intervention independently at home for 15–30 min per session, 3–5 days per week. Intervention activities were integrated into routine caregiving contexts such as feeding, play, and daily care. Caregivers recorded intervention activities and infant responses using standardized logs.

Ongoing supervision, guidance, and individualized feedback were provided remotely via a WeChat-based platform to support adherence and allow adjustment of intervention strategies according to the infant's developmental progress. In addition, outpatient follow-up visits were conducted every three months to monitor language development and caregiver compliance.

The control group received routine outpatient follow-up every 6–8 weeks, which included general health education and brief developmental guidance, but did not receive structured caregiver training, systematic monitoring, or individualized language intervention.

### Assessment

Social communication abilities were evaluated as part of routine developmental assessment conducted by trained clinicians in the outpatient rehabilitation setting. Indicators of social communication included eye contact, social smiling, responsiveness to caregiver vocalization, initiation of interaction, and engagement during play. Classification of weaker social communication abilities was based on standardized developmental assessment results in combination with structured clinical observation and professional judgment, consistent with routine early developmental evaluation practices. In addition to language-related outcomes, comprehensive developmental assessment was conducted using standardized instruments, including the Gesell Developmental Schedules and/or the Chinese version of the Children Neuropsychological and Behavioral Scale (CNBS-R2016), covering cognitive, motor, language, and social–adaptive domains. These assessments were administered in a standardized one-on-one format by trained professionals during outpatient visits.

Significant language improvement was defined as an increase in language developmental quotient (DQ) of at least 15 points (ΔDQ ≥ 15). This threshold corresponds to approximately one standard deviation in commonly used developmental assessment scales and has been widely adopted in early developmental research to indicate clinically meaningful change rather than measurement variability. Similar cutoff values have been used in prior studies evaluating early intervention outcomes in infants and young children.

In addition to language abilities, other developmental domains—including cognitive, motor, and social–adaptive functioning—were assessed using standardized developmental scales administered by trained professionals. Results indicated modest improvements in cognitive and social–adaptive domains in the intervention group, whereas motor development did not differ significantly between groups.

### Outcome measures

The primary outcome was the change in language developmental quotient (DQ) measured at baseline and at 3-month follow-up using validated scales such as the Gesell or CNBS-R2016 language subdomains. Secondary outcomes included the proportion of caregivers with high adherence to daily training (defined as ≥80% completion of daily logs), parental satisfaction (measured by a 10-point Likert scale at follow-up), and the frequency of unplanned developmental consultations or medical visits during the study period.

Feasibility was evaluated using multiple indicators, including parental adherence to the intervention protocol, follow-up completion rates, and caregiver-reported acceptability. The majority of caregivers completed the intervention as prescribed, attrition was low, and parental feedback indicated high acceptability and minimal distress for both caregivers and infants. These findings support the feasibility of implementing a caregiver-mediated, home-based language intervention within routine clinical settings.

### Data collection and statistical analysis

Background data were collected from medical records and caregiver interviews at enrollment, including infant sex, gestational age, birth weight, perinatal complications, and family characteristics such as parental education level, household income, and primary caregiver status. Clinical assessments were performed in person by trained professionals, whereas caregiver-reported information was obtained using structured questionnaires. All clinical data, language scores, and caregiver-reported logs were extracted from the hospital's rehabilitation records and follow-up documentation systems. Descriptive statistics were used to summarize baseline characteristics. Continuous variables were expressed as mean ± standard deviation (SD) and compared using the independent-samples *t*-test or Mann–Whitney *U* test as appropriate. Categorical variables were presented as frequencies and percentages and analyzed using the chi-square test or Fisher's exact test. A logistic regression model was employed to identify independent predictors of significant improvement (defined as an increase in DQ ≥15 points). Statistical significance was set at *p* < 0.05. All analyses were performed using SPSS version 26.0 (IBM Corp., Armonk, NY, USA).

A three-month follow-up interval was selected because early language development during infancy is characterized by rapid change, and previous early intervention studies have demonstrated that measurable improvements in communicative behaviors and developmental quotients can be observed within this time frame. In addition, a three-month interval aligns with routine outpatient follow-up schedules, thereby enhancing feasibility and minimizing attrition.

## Results

### Baseline characteristics of participants

A total of 195 infants diagnosed with early language developmental delay were included in this retrospective analysis, with 83 receiving structured home-based rehabilitation and 112 receiving standard outpatient observation. Baseline demographic and clinical features were largely balanced between the two groups. The average age at enrollment was 8.3 ± 2.1 months in the intervention group and 8.6 ± 2.4 months in the control group (*P* = 0.412). Male proportions were similar (57.8% vs. 55.4%, *P* = 0.737), and no significant differences were observed in gestational age (mean 38.5 vs. 38.3 weeks, *P* = 0.564), birth weight (*P* = 0.473), or Apgar scores (*P* = 0.732).

Pre-intervention language DQ scores were comparable (71.2 ± 6.9 vs. 70.8 ± 7.3, *P* = 0.689), and the rates of comorbid developmental delays (e.g., fine motor delay) were not significantly different. Parental education, household income, and home language environment scores were also statistically matched (see Table 1). This balance ensured a robust comparison of intervention effects.

**Table 1:** Baseline characteristics of infants in the intervention and control groups.

Variable	Intervention group (*n* = 83)	Control group (*n* = 112)	*p*-Value
Age at enrollment (months)	6.4 ± 2.1	6.6 ± 2.3	0.482
Male, *n* (%)	47 (56.6%)	64 (57.1%)	0.942
Gestational age (weeks)	38.1 ± 1.4	38.0 ± 1.3	0.653
Birth weight (g)	3,220 ± 420	3,180 ± 440	0.477
Low birth weight (<2,500 g), *n* (%)	7 (8.4%)	10 (8.9%)	0.902
Preterm birth (<37 weeks), *n* (%)	9 (10.8%)	13 (11.6%)	0.860
Cesarean delivery, *n* (%)	35 (42.2%)	49 (43.8%)	0.821
Exclusive breastfeeding at 3 months	76 (91.6%)	98 (87.5%)	0.394
Parental education ≥college, *n* (%)	58 (69.9%)	76 (67.9%)	0.765
Monthly family income ≥10,000 RMB (%)	41 (49.4%)	55 (49.1%)	0.968
Urban residence, *n* (%)	62 (74.7%)	83 (74.1%)	0.924
Neonatal complications, *n* (%)	12 (14.5%)	15 (13.4%)	0.835
Passed hearing screening, *n* (%)	80 (96.4%)	108 (96.4%)	1.000
Family history of language delay, *n* (%)	5 (6.0%)	7 (6.3%)	0.939
Daily language exposure (hours/day)	4.1 ± 1.3	4.0 ± 1.4	0.624
Primary caregiver = parent, *n* (%)	77 (92.8%)	102 (91.1%)	0.684
Number of siblings	0.63 ± 0.48	0.66 ± 0.52	0.612
Bayley-III cognitive score	90.5 ± 8.7	91.2 ± 9.1	0.591
Bayley-III language score	82.3 ± 9.5	82.7 ± 8.9	0.781
Bayley-III motor score	93.8 ± 7.9	94.1 ± 7.6	0.774

BMI, body mass index; CNS, central nervous system; IQ, intelligence quotient; RMB, renminbi (Chinese yuan); SD, standard deviation; VEGF, vascular endothelial growth factor.

### Language outcomes after 3 months

By the end of the 3-month follow-up period, significant improvements in language development were observed in the intervention group. The average gain in language DQ was +16.4 ± 5.3 points, which was more than double the +7.2 ± 4.9 points seen in the control group (*P* < 0.001). The values “+16.4 ± 5.3 points” and “+7.2 ± 4.9 points” represent the mean (±standard deviation) change in language DQ from baseline to the three-month follow-up in the intervention group and control group, respectively. These values reflect within-group improvements over time rather than absolute post-intervention scores. Furthermore, the proportion of children achieving a post-treatment DQ ≥ 85 was 48.2% in the intervention group compared to just 19.6% in the control group (*P* < 0.001). A meaningful improvement, defined as a ≥15-point increase in DQ, occurred in 72.3% of intervention infants vs. only 28.6% in controls (*P* < 0.001), as shown in Table 2.

**Table 2:** Comparison of intervention outcomes between groups.

Outcome measure	Intervention group (*n* = 83)	Control group (*n* = 112)	*p*-Value
Bayley-III language composite score (mean ± SD)	94.2 ± 10.7	87.5 ± 11.3	<0.001
Language improvement ≥10 points (%)	59 (71.1%)	52 (46.4%)	0.002
Motor composite score (mean ± SD)	96.8 ± 9.3	94.1 ± 10.1	0.048
Cognitive composite score (mean ± SD)	97.5 ± 10.2	93.9 ± 11.4	0.026
Follow-up compliance (%)	78 (94.0%)	79 (70.5%)	<0.001
Parent-reported satisfaction ≥9/10 (%)	75 (90.4%)	76 (67.9%)	<0.001
Missed scheduled sessions (mean ± SD)	0.8 ± 1.1	2.2 ± 2.6	<0.001
Use of teleconsultation tools (%)	65 (78.3%)	31 (27.7%)	<0.001
Parental stress score (PSS-10)	12.4 ± 3.2	15.9 ± 4.7	<0.001

PSS-10, perceived stress scale, 10-item version; SD, standard deviation.

Additionally, expressive vocabulary counts (as recorded by caregivers) increased more significantly in the intervention group (average gain: 45.6 ± 13.2 words vs. 19.8 ± 11.4 words, *P* < 0.001), indicating not only quantitative improvement but also early functional language acquisition.

### Caregiver adherence, participation, and satisfaction

Of the 83 families assigned to home-based training, 65 (78.3%) completed more than 80% of prescribed daily training activities, with 14 (16.9%) completing between 60% and 80%, and only 4 (4.8%) performing below 60%. Reasons for lower adherence included caregiver fatigue and competing household responsibilities. Weekly follow-up calls revealed high engagement, and no major implementation barriers were recorded.

Caregivers in the intervention group reported significantly greater satisfaction with the rehabilitation experience (9.1 ± 0.6 vs. 7.3 ± 1.2, *P* < 0.001). Qualitative feedback indicated that caregivers valued the structured guidance, real-time feedback, and perceived benefit in child responsiveness. As detailed in Table 3, over 92% of caregivers in the intervention group rated the experience as “very helpful,” compared to only 58% in the control group who rated their passive observation approach positively.

**Table 3:** Changes in developmental scores before and after intervention within groups.

Outcome measure	Intervention group (*n* = 83) Pre	Outcome measure	Intervention group (*n* = 83) Pre	Intervention group post	*p*-value (Within Group)	Control Group (*n* = 112) Pre	Control Group Post	*p*-value (Within Group)	*p*-value (Between Groups, Post)
Bayley-III language composite score	78.2 ± 5.9	Bayley-III Language Composite Score	78.2 ± 5.9	85.6 ± 6.4	<0.001	79.1 ± 6.1	81.0 ± 6.5	0.032	<0.001
Bayley-III cognitive composite score	85.7 ± 6.2	Bayley-III Cognitive Composite Score	85.7 ± 6.2	89.1 ± 6.3	<0.001	86.2 ± 6.4	87.5 ± 6.0	0.048	0.013
Bayley-III motor composite score	90.3 ± 5.8	Bayley-III Motor Composite Score	90.3 ± 5.8	91.8 ± 6.1	0.056	89.7 ± 5.5	90.1 ± 5.8	0.431	0.071
Bayley-III Social-Emotional Score	92.1 ± 4.9	Bayley-III Social-Emotional Score	92.1 ± 4.9	94.6 ± 5.0	0.004	91.6 ± 5.1	92.2 ± 5.3	0.237	0.008
Bayley-III adaptive behavior composite	87.3 ± 6.0	Bayley-III Adaptive Behavior Composite	87.3 ± 6.0	90.4 ± 6.2	0.009	87.9 ± 6.1	88.5 ± 6.3	0.375	0.027

### Health resource utilization and adverse events

Fewer unplanned outpatient visits were noted in the intervention group during follow-up (6/83, 7.2%) compared to the control group (18/112, 16.1%, *P* = 0.048). These visits primarily addressed parental concerns about delayed progress, feeding, or sleep disturbances. Importantly, no serious adverse events were observed in either group. Minor caregiver-reported fatigue or emotional strain was documented in 11 cases but resolved spontaneously. These findings suggest that home-based interventions were well-tolerated and may reduce secondary healthcare burden (Table 4).

**Table 4:** Parental satisfaction and intervention compliance between groups.

Outcome indicator	Intervention group (*n* = 83)	Control group (*n* = 112)	*p*-value
High parental satisfaction (score ≥9/10), *n* (%)	74 (89.2%)	73 (65.2%)	<0.001
Full adherence to intervention schedule, *n* (%)	68 (81.9%)	56 (50.0%)	<0.001
Number of missed sessions [median (IQR)]	1 [0–2]	3 [1–5]	<0.001
Parent-reported ease of training (≥4/5), *n* (%)	71 (85.5%)	63 (56.3%)	<0.001
Number of home training sessions completed	38.7 ± 6.1	29.4 ± 7.9	<0.001

### Multivariate analysis: predictors of language recovery

To explore factors independently associated with significant language improvement (defined as ΔDQ ≥ 15), a multivariate logistic regression model was constructed. Variables entered into the model included baseline DQ, presence of sibling interaction, frequency of parent–child verbal interaction, maternal education level, home language stimulation score, and group assignment.

Participation in the home-based rehabilitation group emerged as the strongest predictor (OR = 3.91, 95% CI: 2.02–7.58, *P* < 0.001). Additional predictors included frequent verbal engagement (≥3 structured interactions/day, OR = 1.92, *P* = 0.037) and baseline DQ > 65 (OR = 2.14, *P* = 0.024). Maternal education, income level, and birth-related variables were not significantly associated with recovery after adjusting for confounders. Detailed coefficients and odds ratios are presented in Table 5.

**Table 5:** Multivariate logistic regression for predictors of language developmental delay at 6 months.

Variable	Odds ratio (OR)	95% confidence interval (CI)	*p*-Value
Home-based rehabilitation (yes vs. no)	0.41	0.22–0.76	0.005
Male sex	1.34	0.72–2.48	0.347
Gestational age <37 weeks (prematurity)	2.63	1.29–5.34	0.007
Birth weight <2.5 kg (low birth weight)	2.25	1.17–4.32	0.015
Firstborn status (yes vs. no)	1.09	0.58–2.05	0.792
Cesarean section delivery (yes vs. no)	1.45	0.78–2.69	0.237
Maternal age ≥35 years	1.68	0.89–3.16	0.108
Maternal education ≤high school	2.08	1.12–3.86	0.021
Family income <5,000 RMB/month	1.73	0.93–3.22	0.084
History of neonatal ICU admission	2.41	1.13–5.12	0.023
Delayed gross motor milestones (per baseline DDST)	2.48	1.28–4.81	0.007
Poor parental compliance (<80% rehabilitation adherence)	2.89	1.51–5.52	0.001
Absence of caregiver stimulation ≥1 h/day	2.17	1.14–4.12	0.018
Urban residence (vs. rural)	0.78	0.42–1.44	0.425
Presence of coexisting vision or hearing concerns	2.97	1.31–6.72	0.009

CI, confidence interval; DDST, denver developmental screening test; ICU, intensive care unit; OR, odds ratio; RMB, Renminbi (Chinese Yuan).

## Discussion

This retrospective study evaluated the feasibility and efficacy of a structured home-based rehabilitation program for language delay in infants aged 0–1 year during the COVID-19 pandemic. Our findings suggest that personalized, caregiver-delivered early intervention programs can significantly enhance language development outcomes within a relatively short time frame, even in the absence of frequent in-person therapy.

The primary finding of this study is the significant improvement in developmental quotient (DQ) among infants who received home-based intervention, compared to those under routine observation. Specifically, over 70% of children in the intervention group achieved meaningful gains (ΔDQ ≥ 15), and nearly half reached a normalized DQ ≥ 85 after three months. These improvements align with prior studies indicating that early neuroplasticity is particularly responsive to enriched language environments and targeted stimulation during infancy (2, 12, 13). Our results also echo international findings from parent-led models such as the Hanen Program and “It Takes Two to Talk,” which emphasize the efficacy of coaching parents to serve as primary intervention agents (14, 15). However, our study extends this evidence to infants younger than 12 months—an age group often excluded from formal interventions due to concerns about limited engagement capacity. By equipping caregivers with structured materials, remote feedback, and daily activity schedules, we demonstrated that even very young children can benefit from early, non-specialist-delivered intervention. From a practical standpoint, the intervention was both acceptable and sustainable. Adherence rates exceeded 75%, and satisfaction levels among caregivers were high. These findings are notable given the increased family stress and healthcare access limitations. Remote supervision, simplified tools, and regular feedback likely contributed to high engagement, as did the use of mobile technology for monitoring and encouragement. Importantly, no serious adverse events or caregiver burden-related dropout was observed, affirming the safety and practicality of this model in real-world settings.

Health system implications are also important to highlight. The home-based group had significantly fewer unplanned outpatient visits and showed better engagement metrics, suggesting that such programs may help reduce resource burden in overextended pediatric and rehabilitation clinics. In countries with workforce shortages or limited access to early intervention services, home-based rehabilitation may serve as a scalable, cost-effective alternative (16–18). Nonetheless, this study has limitations. As a single-center retrospective cohort study, it is subject to potential confounding despite efforts to match baseline characteristics. Language development was assessed using DQ scores rather than more granular, language-specific tools, and the short follow-up period did not permit assessment of long-term language trajectories. Additionally, adherence was self-reported, introducing the possibility of reporting bias.

In addition to effectiveness, the present study provides evidence regarding the feasibility of implementing a caregiver-mediated, home-based early language intervention in routine clinical settings. Feasibility was reflected by high parental acceptability and adherence, low attrition during follow-up, and good tolerability of the intervention. Most caregivers were able to integrate the intervention into daily routines and complete the prescribed activities, and no intervention-related distress or adverse events were reported for either infants or caregivers. Although formal qualitative assessment of emotional experiences was not conducted, caregiver feedback and sustained participation suggest that the program was acceptable and manageable. Together, these findings support the feasibility of this home-based intervention model and justify further evaluation in prospective and larger-scale studies.

Future research should include randomized controlled trials with longer follow-up periods and more diverse populations. It would also be valuable to integrate digital tools such as AI-assisted monitoring, speech-recognition feedback, and video modeling to further support families and enhance interactivity. Moreover, qualitative exploration of caregiver experiences could provide deeper insight into motivational barriers and optimal delivery models for home-based care.

## Conclusion

This study demonstrates that a structured, home-based rehabilitation program is both feasible and effective in improving early language development among infants aged 0–1 year with suspected language delays during the pandemic. Infants who received targeted caregiver-guided interventions showed significantly greater improvements in developmental quotient (DQ) scores and were more likely to reach age-appropriate language milestones compared to those receiving routine care. The program was well tolerated, with high adherence and caregiver satisfaction, and it reduced unnecessary outpatient utilization. These findings suggest that home-based language interventions, when properly structured and supervised, can serve as a practical alternative or complement to traditional in-clinic therapy—especially in contexts of limited access, public health emergencies, or early-stage developmental risk. Future prospective and multi-center trials are warranted to further validate this approach and explore its long-term developmental and social benefits.

## Data Availability

The original contributions presented in the study are included in the article/Supplementary Material, further inquiries can be directed to the corresponding author.
